# Intra-arterial hepatic beads loaded with irinotecan (DEBIRI) with mFOLFOX6 in unresectable liver metastases from colorectal cancer: a Phase 2 study

**DOI:** 10.1038/s41416-020-0917-4

**Published:** 2020-06-08

**Authors:** Simon Pernot, Olivier Pellerin, Pascal Artru, Carole Montérymard, Denis Smith, Jean-Luc Raoul, Christelle De La Fouchardière, Laetitia Dahan, Rosine Guimbaud, David Sefrioui, Jean-Louis Jouve, Côme Lepage, David Tougeron, Julien Taieb, for FFCD1201-DEBIRI investigators/Collaborators

**Affiliations:** 1 Département de Gastroentérologie et d’Oncologie Digestive, Hôpital Européen George Pompidou, Paris, France; 2 Service de Radiologie, Hôpital Européen Georges Pompidou, Paris, France; 3 Institut de Cancérologie, Hôpital Privé Jean Mermoz, Lyon, France; 4 Département Biostatistiques, Fédération Francophone de Cancérologie Digestive, Dijon, France; 5 EPICAD INSERM LNC-UMR 1231, Université de Bourgogne Franche Comté, Dijon, France; 6 Service d’Oncologie, CHU Bordeaux Hôpital St. André, Bordeaux, France; 7 Département d’Oncologie Médicale, Institut Paoli Calmettes CAC, Marseille, France; 8 Service de Médecine, Centre Léon Bérard, Lyon, France; 9 Service d’HGE et d’Oncologie, CHU La Timone, Marseille, France; 10 Service d’HGE, CHU Toulouse Rangueil, Toulouse, France; 11 Hôpital Universitaire de Rouen, Normandie Université, Service d’Hépato-gastroentérologie, UNIROUEN, Inserm 1245, IRON Group, 76000 Rouen, France; 12 Service d’HGE, CHU Le Bocage (Dijon), Dijon, France; 13 Service d’Oncologie, CHU Poitiers, La Milétrie, Poitiers, France

**Keywords:** Metastasis, Cancer therapy, Colorectal cancer, Drug delivery

## Abstract

**Background:**

Chemo-embolisation with drug-eluting beads loaded with irinotecan (DEBIRI) increased survival as compared with intravenous irinotecan in chemorefractory patients with liver-dominant metastases from colorectal cancer (LMCRC). First-line DEBIRI with systemic chemotherapy may increase survival and secondary resection.

**Methods:**

In the FFCD-1201 single-arm Phase 2 study, patients with untreated, non-resectable LMCRC received DEBIRI plus mFOLFOX6. Four courses of DEBIRI were performed alternating right and left lobe or two sessions with both lobes treated during the same session.

**Results:**

Fifty-seven patients were enrolled. Grade 3–5 toxicities were more frequent when both lobes were treated during the same session (90.5% versus 52.8%). Nine-month PFS rate was 53.6% (95% CI, 41.8–65.1%). The objective response rate (RECIST 1.1) was 73.2%, and the secondary R0 surgery was 33%. With a median follow-up of 38.3 months, median OS was 37.4 months (95% CI, 25.7–45.8), and median PFS 10.8 months (95% CI, 8.2–12.3).

**Conclusions:**

Front-line DEBIRI + mFOLFOX6 should not be recommended as the hypothesised 9-month PFS was not met. However, high response rate, deep responses, and prolonged OS encourage further evaluation in strategies integrating biologic agent, in particular in patients with secondary surgery as the main goal.

**Clinical trial registration:**

NCT01839877.

## Background

Over 80% of patients with liver metastases from colorectal cancer (LMCRC) present with unresectable disease. The intensification of first-line chemotherapy in unresectable patients allows a significant rate of secondary resection and led to an increased survival.^1–3^ Intrahepatic arterial delivery of chemotherapy has been proposed in patients with liver-only disease to treat tumour cells with high local concentrations of anticancer agents and decreased systemic toxicity.

Transarterial chemo-embolisation (TACE) is a standard treatment for hepatocellular carcinoma and has for many years been used to treat LMCRC, albeit with a lack of prospective and comparative studies. Most reports are of small retrospective studies with various chemotherapy regimens and embolisation procedures in heterogeneous patient populations.^4^ Intra-arterial hepatic administration of drug-eluting beads (DEB) loaded with chemotherapy drugs have been developed to standardise the embolisation procedure compared to conventional TACE with use of calibrated non-resorbable beads, which can load and continuously release cytotoxic drugs into the target tissues.

In a Phase 3 trial on 74 patients,^5^ DEB loaded with irinotecan (DEBIRI) plus intravenous 5-fluoro-uracil (5FU) was compared to intravenous 5FU plus irinotecan (Folfiri) in patients with unresectable LMCRC after failure of at least two lines of treatment. Overall survival (OS), progression-free survival (PFS), and response rate significantly improved in the DEBIRI arm. However, heavily pretreated patients with liver-limited disease (LLD) are not frequent and the rate of secondary resectability is very low in this subgroup of patients. Use of DEBIRI before surgery also increases the histological response, as suggested by the PARAGON II study,^5^ and thus may potentially reduce the risk of recurrence.^6^

We hypothesise that upfront use of DEBIRI combined with systemic chemotherapy in liver-dominant metastatic colorectal cancer (mCRC) patients could improve treatment efficacy, limit systemic toxicity, and thus improve survival and secondary resectability. Our prospective, multi-centre, single-arm, Phase 2 study evaluated the feasibility, safety, and efficacy of a conventional systemic chemotherapy regimen 5FU plus oxaliplatin (FOLFOX) combined with intra-arterial hepatic DEBIRI as first-line treatment of mCRC patients with liver-dominant mCRC.

## Methods

### Study design

This multi-centre, single-arm, Phase 2 study was approved by the French ethics committee “CCP Ile de France 8” (No. 1212113). All patients provided informed consent before study enrolment. The main eligibility criterion was previously untreated mCRC with unresectable liver metastases, as defined at a local multidisciplinary team meeting. Main exclusion criteria included liver involvement >60% or impaired hepatic function and extrahepatic metastases on computed tomographic (CT) scan, except lung nodules if <4 and <1 cm each. Inclusion/exclusion criteria are detailed in Supplementary Information 1. Concomitant administration of any targeted therapies was not permitted, considering that toxicity of the combination of DEBIRI plus anti-vascular endothelial growth factor (anti-VEGF) or anti-epidermal growth factor receptor (anti-EGFR) is unknown (no Phase 1 study available), and the biliary or vascular cumulative toxicity that have been reported in other trials with hepatic arterial chemotherapy.^7^

### Procedures

Patients received induction chemotherapy with FOLFOX: oxaliplatin 85 mg/m^2^ as a 2-h infusion at day 1, leucovorin 400 mg/m^2^ as a 120-min infusion at day 1 followed by 5FU 400 mg/m^2^ bolus at day 1 and 2400 mg/m^2^ 46-h continuous 5FU infusion, 1 cycle every 2 weeks. Patients received treatment with DC Bead LUMI™ 100–300 (Biocompatibles UK limited) loaded with irinotecan 50 mg/ml, 1 vial per lobe and per treatment (meaning 1 vial in case of unilobar administration, and 2 vials in case of bilobar administration). Each treatment session was performed 48–72 h after a chemotherapy cycle. Treatment administration was performed using a unilateral femoral approach. Depending of patient case and according to the choice of the investigators, the treatment could be administered in a bilobar approach or a sequential unilobar approach: in patients treated with a bilobar approach, both lobes were treated at each session after the second and fourth chemotherapy cycles; in patients treated with a sequential unilobar approach, only one lobe was treated per session, each lobe being treated alternately, after the second, third, fourth, and fifth cycles of chemotherapy. After a preplanned safety analysis performed after 27 patients were treated, the safety board recommendation was to treat patients with the sequential unilobar approach because of better tolerability. The procedure and periprocedural medication are described in Supplementary Information 2 and 3.

Prophylaxis with granulocyte-colony stimulating factor (G-CSF) could be used as primary prophylaxis, at the investigator’s discretion. Patients continued treatment until unacceptable toxicity, disease progression, consent withdrawal, or investigator choice.

### Endpoints/statistical analysis

The primary endpoint was the rate of PFS at 9 months, according to the local investigator evaluation. A one-step Fleming plan was used, with an *α* risk of 5% and unilateral power (1 − *β*) of 90%, testing the following assumptions: *H*_0_: 55% of patients alive without progression at 9 months is uninteresting; *H*_1_: 75% is expected. Taking into account a rate of 20% of patients lost to follow-up (without evaluation during the first 9 months of treatment), 58 patients had to be included.

Secondary endpoints included safety (according to National Cancer Institute Common Terminology Criteria for Adverse Events (NCI-CTCAE) v4.0), objective response rate (ORR) according to response evaluation criteria in solid tumours (RECIST) 1.1 criteria, PFS, OS, secondary resectability rate, depth of response and early tumour shrinkage at 8 weeks; definitions are provided in Supplementary Information 4.

All adverse events were graded according to the NCI-CTCAE version 4.0. All patients with LMCRC who received at least one dose of treatment were included in the analysis of safety population. An interim safety analysis was planned after treatment of 27 patients, and an independent safety committee board was implemented.

The primary endpoint was analysed in a modified intention-to-treat (mITT) population defined as all patients who fulfilled eligibility criteria and has received at least one session of chemo-embolisation, at least one dose of chemotherapy, and who had at least one radiological evaluation during the 9 months of follow-up. Secondary endpoint was analysed in the ITT population.

Exploratory analyses to determine prognostic factors of PFS and OS were performed using univariate and multivariate Cox proportional hazards models. Hazard ratios and 95% confidence interval (CI) were estimated. Variables with *p* < 0.20 in univariate analyses were used in multivariate analyses.

All data were reported using the usual descriptive statistics: qualitative variables are described with percentages and quantitative variables with mean, standard deviation, median, interquartile interval (Q1–Q3), and range (minimum–maximum). Analyses were done using SAS 9.4 (SAS Institute, Cary, NC).

## Results

### Patient characteristics

From May 2013 to December 2016, 58 patients were included and treated in 10 participating centres in France. Diagnosis of LMCRC was reconsidered and requalified as LM from pancreatic adenocarcinoma after inclusion in one patient, who was excluded from the final analysis. One patient was not analysed in the mITT population because he had no evaluation after the treatment during the study period (9 months). Patient characteristics are described in Table 1. Briefly, all patients had synchronous metastatic disease with primary tumour removed in 10 patients, including one with preoperative radio-chemotherapy for rectal cancer. Patients had bilobar metastatic disease in 88% of cases and a mean 9 LM (range 1–20). Lung nodules were described in 12% of patients on CT scan in respect of inclusion criteria.

**Table 1:** Patient and treatment characteristics.

	*N* = 57
Median age (min.–max., years)	63 (44–78)
Gender (*n*, %)	
Female	25 (44)
Male	32 (56)
ECOG-PS (*n*, %)	
0	24 (42.1)
1	30 (52.6)
2	3 (5.3)
Median number of LM (min.–max.)	9.5 (1–20)
Distribution of LM (*n*, %)	
Right lobe only	5 (8.8)
Left lobe only	2 (3.5)
Bilobar disease	50 (87.7)
Lung metastases on CT scan (*n*, %)	7 (12.3)
Molecular status (*n*, %)	
Ras mutated	30 (52.6)
BRAF mutated	2 (3.5)
RAS/BRAF wild type	23 (40.4)
ND	2 (3.5)
CEA level (*n*, %)	
≤5 ULN	18 (31.6%)
>5 ULN−≤15 ULN	16 (28.1%)
>15 ULN	23 (40.4%)
Administration of DEBIRI (*n*, %)	
Unilobar administration	36 (63.2)
Bilobar administration	21 (36.8)
Sidedness of primary tumour (*n*, %)	
Rectum	11 (19.3)
Left colon	34 (59.6)
Right colon	12 (21.1)

### Treatment compliance

All patients received at least one session of DEBIRI. The full DEBIRI treatment plan (2 or 4 sessions) was performed in 49% of patients. Bilobar administration was performed in 36.8% of patients, and a sequential unilobar administration was performed in 63.2%. Six patients planned for 2 bilobar sessions received only one bilobar administration instead of 2 due to grade 3–4 toxicity after the first session. The median number of FOLFOX cycles was 8 (range 2–28). The median dose intensities (all cycles of FOLFOX) were 92.4% for oxaliplatin, 80% for 5FU bolus, and 97.8% for continuous 5FU infusion. Twenty-eight patients (49%) received G-CSF.

### Efficacy

**Fig. 1:**
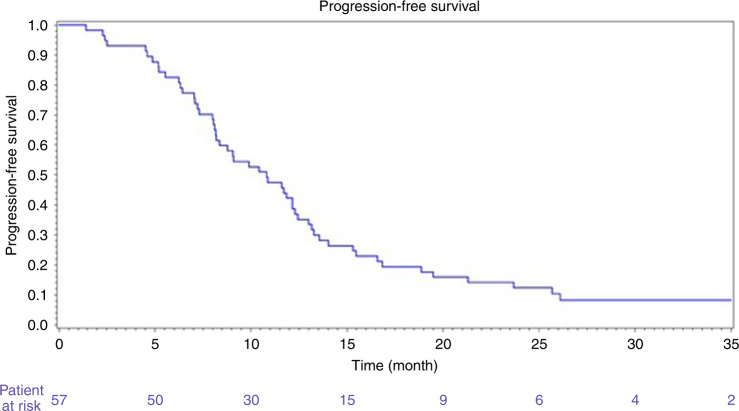
Progression-free survival. *Y* = probability of PFS.

**Fig. 2:**
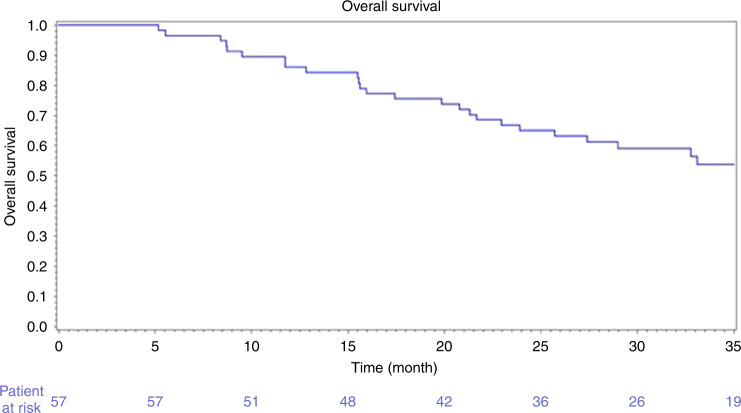
Overall survival. *Y* = probability of survival.

The 6- and 9-month PFS rates according to investigators were 82.4% [95% CI 69.8–90.1] and 53.6% [95% CI 41.8–65.1], respectively, in mITT population. After a median follow-up of 38.3 months [95% CI 32.2–41.9], 4 patients were alive without progression in ITT population. Median PFS was 10.8 months [95% CI 8.2–12.3] (Fig. 1) and median OS was 37.4 months [95% CI, 25.7–45.8] (Fig. 2). Median-specific liver PFS was 10.9 months [95% CI, 8.2–12.4]. Post-progression treatments were available in 49 patients. After progression, patients received a median of two treatments (Supplementary Table S1).

A blind central review assessment for the primary objective was made by a single independent radiologist. Results were concordant with investigator assessment, with a median PFS of 10.4 months [95% CI 7.2–13.6].

**Fig. 3:**
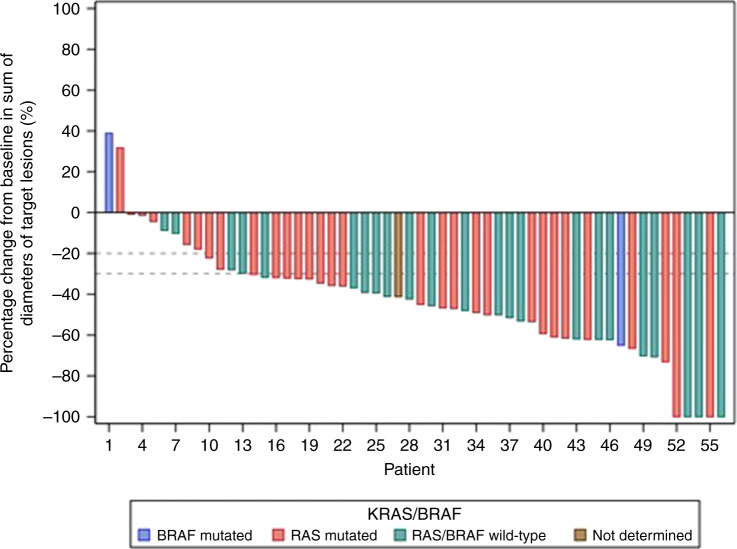
Depth of response. Waterfall plot of change from baseline in sum of diameters of target lesions (%).

According to investigator evaluation, an ORR was observed in 41 patients (73.2%) including 4 complete responses. Disease control rate (DCR) was 92.9%. The median depth of response was −43.6% (Q1: −61.7; Q3: −31) (Fig. 3). The rate of early tumour shrinkage was 52.1% [95% CI 37.2–66.7].

### Secondary resection or ablative treatment

Nineteen patients (33%) underwent R0 resection of LM; for this subgroup, median PFS was 13 months [95% CI 8.8; 16.6] and median OS was not reached. OS rate at 2 and 3 years were 94.74% [95% CI 68.12; 99.24] and 75.49% [95% CI 45.80; 90.37], respectively. Of the 17 patients with recurrence after curative intent surgery, 11 were eligible for a second curative intent surgery/ablation. Altogether, 8 of these 19 patients were alive with no evidence of disease after a median follow-up of 3.7 years (range 2.5–4.7).

**Fig. 4:**
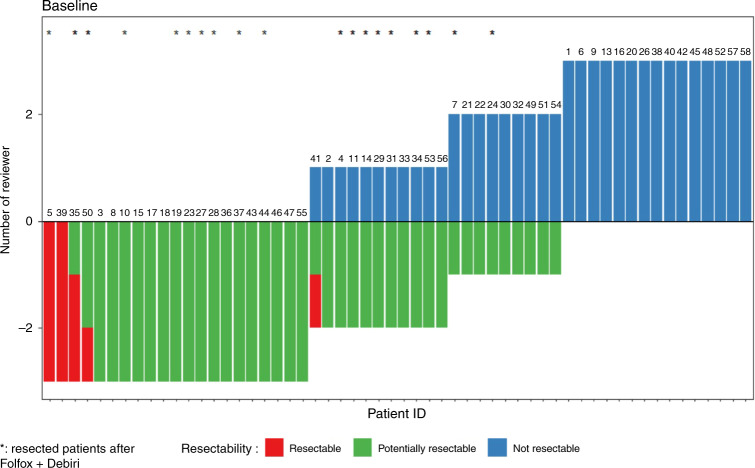
Centralised review of resectability at baseline. Post hoc evaluation of resectability based on a CT scan at baseline for each patient, by three independant experienced hepatic surgeons. *Resected patients after Folfox + Debiri.

A post hoc independent evaluation of resectability by three experienced hepatic surgeons was performed based on a CT scan at baseline. According to this centralised review, 53/56 patients (94.6%) were retrospectively confirmed as non-resectable by at least 2 surgeons (Fig. 4). Two patients were considered resectable at baseline (3.5%) by the 3 surgeons and 1 by 2 of the 3 surgeons.

### Prognostic factors

The effect on survival of clinical and laboratory findings and unilobar versus bilobar administration was assessed. *BRAF* mutation was the only factor associated with worse PFS and OS in both univariate and multivariate analysis. Primary tumour location and administration modality were not prognostic (Supplementary Tables S2 and S3).

### Safety

One toxic death possibly related to DEBIRI was reported (peritonitis). The main grade 3–4 toxicities were neutropenia (24.6%), diarrhoea (12.3%), abdominal pain (14%), and pancreatitis/cholecystitis (8.8%/5.3). Importantly, almost all G3/4/5 toxicities were more frequent with the bilobar approach than the unilobar approach (87.5% versus 47.2%; Table 2). Adverse events are detailed in Supplementary Table S4. Two patients stopped chemotherapy due to major toxicity after 2 and 6 cycles of FOLFOX, respectively. A delay in administration of chemotherapy due to toxicity was reported in 16 patients.

**Table 2:** Grade 3/4/5 adverse events in overall population and according to the treatment modality.

Toxicity		Grade 3/4/5 toxicity	
	All, *N* = 57	Unilobar, *N* = 36	Bilobar, *N* = 21
All	38 (66.7)	19 (52.8)	19 (90.5)
Non-hematologic			
Any non-hematologic	27 (47.4)	12 (33.3)	15 (71.4)
Asthenia	3 (5.3)	0	3 (14.3)
Hypertension	1 (1.8)	0	1 (4.8)
Any gastrointestinal	20 (35.1)	9 (25.0)	11 (52.4)
Nausea	3 (5.3)	1 (2.8)	2 (9.5)
Vomiting	3 (5.3)	3 (8.3)	0
Diarrhoea	7 (12.3)	3 (8.3)	4 (19.0)
Abdominal pain	8 (14.0)	2 (5.6)	6 (28.6)
Small bowel obstruction	2 (3.5)	1 (2.8)	1 (4.8)
Any extrahepatic perfusion	7	2 (5.6)	5 (23.8)
Peritonitis	1 (1.8)	0	1 (4.8)
Pancreatitis	5 (8.8)	1 (2.8)	4 (19)
Cholecystitis	3 (5.3)	2 (5.6)	1 (4.8)
Hematologic	20 (35.1)	11 (30.6)	9 (42.9)
Anemia	3 (5.3)	2 (5.6)	1 (4.8)
Thrombocytopenia	3 (5.3)	0	3 (14.3)
Lymphopenia	2 (3.5)	2 (5.6)	0
Leucopenia	1 (1.8)	1 (2.8)	0
Neutropenia	14 (24.6)	6 (16.7)	8 (38.1)
Febrile neutropenia	3 (5.3)	2 (5.6)	1 (4.8)

Periprocedural adverse events (occurring during the first 24 h after DEBIRI) of any grade was observed in 75.4% of patients. Post-embolisation syndrome was the most frequent adverse event, with significant abdominal pain (visual analogue scale >3) occurring in 75.4% of patients, nausea/vomiting in 22.8% of patients, and fever in 5.3% of patients. Cardiovascular side effects were not rare, with acute hypertension in 19.3% of patients, thoracic pain in 3 patients, among which 1 was identified as coronary spasm with transitory elevation of troponin, and 2 tachycardia (Supplementary Table S5, online only).

## Discussion

We demonstrated that combination of DEBIRI plus FOLFOX is feasible in LLD mCRC, leading to a high ORR of 73.2% and allowing secondary resection in one-third of patients. Moreover, prolonged OS was observed, with a median of 37.4 months. However, our study did not meet the prespecified primary endpoint, as the 9-month PFS rate was under 75% expected (53.6%). A posteriori, 75% appears challenging considering recent trials with comparable populations in LMCRC.^8^ Nevertheless, a long OS may have been favoured by the depth of response and early tumour shrinkage,^9^ and the subsequent treatment lines considering the spare of targeted agent, and limited irinotecan systemic release^10^ with Folfox + DEBIRI. Finally, while the toxicity profile was manageable, DEBIRI + FOLFOX may lead to serious and specific side effects when two lobes are treated during the same session, but the safety profile was better when DEBIRI was administered in the hepatic lobes one by one.

Doublet chemotherapies with a targeted agent led to an ORR between 33% and 53%, median PFS from 6.8 to 9.2 months, and median OS from 15.1 to 25.8 months.^1–3,11^ ORR reached 55–62% in recent trials in *RAS* wild-type patients only.^12,13^ Compared to doublet chemotherapies, FOLFOXIRI +/− bevacizumab significantly increased ORR and median PFS in patients with non-resectable LMCRC,^1–3,14^ ranging from 43% to 80% and 9.8 to 12.3 months, respectively. The ORR and PFS reported in our study therefore seem in line with those observed in patients treated with intensive systemic regimens such as FOLFOXIRI +/− bevacizumab. Median OS was 37.4 months compared to 25–29 months with the use of triplet +/− bevacizumab.^1–3,14^ However, we have to point that the trials studying triplet included unselected non-resectable mCRC patients, while our study included only liver-dominant patients. Nevertheless, our survival results seem promising in mCRC patients with liver-dominant disease, even though the primary 9-month PFS endpoint was not reached.

Reported secondary resection rates are around 15% with doublet CT + anti-VEGF or anti-EGFR^15,16^ and 23–60% in patients with exclusive LMCRC.^11,14–17^ However, in previous studies of LMCRC, secondary resectability may have been overestimated owing to a significant proportion of patients may actually have been resectable upfront. Indeed, in most of these studies, non-resectability at baseline was defined by the number of metastases >4 and/or size >5 cm and/or by the presence of bilobar metastases or no extrahepatic metastases. However, it is now accepted that these criteria no longer apply.^18–21^ The modern definition of resectability includes the potential for complete resection with tumour-free margins; preservation of viable vascular inflow, outflow, and biliary drainage; and a future minimal remnant liver volume of 30%.^6,22^ Interestingly, in the CELIM study and the FIRE-3 study, retrospective assessment of baseline resectability found that 30% and 22% of patients were technically resectable initially.^15,23^ Our non-resectability assessment before inclusion did not use these size/number criteria and was made by the local multidisciplinary team meeting and 95% of the patients were retrospectively confirmed as non-resectable at baseline by an independent committee of three experienced liver surgeons. Therefore, the resectability rate of 33% is solid and compared favourably with the other studies.

Other intensified hepatic intra-arterial strategies have been or are currently being investigated. Radio-embolisation (selective internal radiation therapy (SIRT)) combined with FOLFOX failed to increase ORR, OS, PFS, or the resection rate, which remained <15% in the FOXFIRE GLOBAL large Phase 3 trial that included patient with liver-dominant disease.^8^ In this study, inclusion criteria and design were very close from our study, and this trial provides recent data in a very similar population and with an alternative transarterial approach and the same systemic regimen (despite that biologic agent could be added after the induction treatment in FOXFIRE GLOBAL trial according to the investigator choice). In this trial, FOLFOX + SIRT led to a median PFS of 11 months and a response rate of 72.4%, similar to those observed with FOLFOX + DEBIRI. The resection rate remained <15% and median OS was only 22.6 months, contrasting with the 33% and 38.3 months reported in our study, which may be explained in part by the depth of response and the early tumour shrinkage observed with DEBIRI, rather than by the liver-specific PFS, which was lower with DEBIRI than with SIRT.

Hepatic arterial infusion of floxuridine chemotherapy showed a response rate of 92% and conversion to resection in 47% of patients^24^ in a Phase 1 trial, but with some FUDR-related complications and biliary toxicity. Hepatic arterial infusion of oxaliplatin combined with systemic chemotherapy and targeted therapy is currently being tested in the PRODIGE 49 Phase 3 trial (NCT02885753). Compared to the latter, DEBIRI has the advantage of being an easy, accessible, and reproducible procedure, as TACE is used worldwide. When first-line DEBIRI in combination with FOLFOX +/− bevacizumab was assessed and compared with systemic chemotherapy alone, ORR increased with DEBIRI, as did resection rate (35% versus 6% [*p* = 0.05]).^25^ However, response was evaluated using the modified RECIST criteria, which are not adequate for DEBIRI in mCRC.^26^ When considering RECIST 1.1 criteria, this study found a response rate of 97% with DEBIRI + FOLFOX, but, more surprisingly, also 95% in patients treated with FOLFOX alone, a value far from those generally reported with this regimen.

We observed a high rate of grade 3–5 adverse events with one toxic death possibly due to DEBIRI. Most frequent toxicities were gastrointestinal and the consequence of extrahepatic perfusion. Interestingly, these specific toxicities, as well as non-specific toxicities, were notably more frequent after DEBIRI was used in a bilobar approach, whereas efficacy parameters were not affected by the bilobar or unilobar approach. This observation during a planned interim safety analysis led to the recommendation to treat patients preferentially with a unilobar approach after inclusion of 27 patients. The reported rate of post-embolisation syndrome in the literature is quite difficult to analyse due to the large heterogeneity in monitoring and reporting of this side effect, ranging from 6% to 100%.^27^ The post-embolisation syndrome rate still seemed high in our trial compared to previous studies. The timing of the procedure with respect to chemotherapy may be involved, since cumulative toxicities of chemotherapy followed by chemo-embolisation could have occurred. The use in front line could also be a reason to explain the higher rate of embolisation syndrome, compared to previous reports in late-line treatment in LMCRC. Indeed, it has been suggested that a majority of patients have a tumour load in late line remaining below their initial tumour load,^28^ and highest tumour load have been associated with more frequent post-embolisation syndromes.^29^

The addition of DEBIRI appears to have had limited impact on the administration of chemotherapy. Indeed, only two patients stopped chemotherapy due to unacceptable toxicity during the induction period. Nevertheless, the median number of cycles of FOLFOX was only eight, suggesting that a large part of patients had a stop-and-go strategy with early maintenance with 5FU or even a break from chemotherapy. Such strategy may have been favoured by the increased toxicity during the induction period.

The strength of our study is that it was conducted in patients carefully selected with non-resectable LMCRC, as confirmed by our panel expert, and was multicentric. Nevertheless, these results need confirmation in a randomised study compared with standard protocols, and in particular with other intensified regimens as triplet chemotherapy. As specified above, we decided to avoid targeted therapy, and FOLFOX + DEBIRI deserves to be tested in combination with targeted therapy. Another weakness is that we did not plan oxaliplatin continuation after the induction period, and some patients had a stop-and-go strategy with early maintenance with 5FU or even a break from chemotherapy, when others kept oxaliplatin until limiting toxicity. This heterogeneity may have impaired the evaluation of PFS.

In conclusion, although the primary endpoint was not met, front-line FOLFOX + DEBIRI without any targeted agent is feasible. Indeed, despite relevant toxicity, the unilobar approach showed a manageable tolerability profile and allows an excellent DCR in non-resectable LMCRC with deep responses, leading to resection in one-third of patients and prolonged survival. Induction treatment with FOLFOX + DEBIRI cannot be considered standard in unresectable patients as the present trial did not meet the prespecified primary endpoint. However, considering a promising response rate and OS, its optimal place in the therapeutic strategy must now be defined by further studies including biologic agents and a more selected patient population, possibly with secondary surgery as main goal, and should be used in a unilobar approach only.

## Supplementary information

Appendix

## Data Availability

Data can be found based on reasonable demand to the promoter “Fédération Française de Cancérologie digestive (FFCD)”.
