# Supplementary material for: Intra-arterial hepatic beads loaded with irinotecan (DEBIRI) with mFOLFOX6 in unresectable liver metastases from colorectal cancer: a Phase 2 study
Source: Br J Cancer. 2020 Jun 8;123(4):518–24. doi: 10.1038/s41416-020-0917-4 (PMC7435188; doi:10.1038/s41416-020-0917-4)
Supplement: Supplementary file 1 — Appendix [file 41416_2020_917_MOESM1_ESM.docx]

Supplementary information

1. **Eligibility criteria**

*Inclusion:*

- Patients over 18 years of age
- Medical insurance coverage
- ECOG PS ≤ 2,
- Life expectancy ≥3 months
- Histologic proof of colorectal adenocarcinoma, and radiologic or histologic proof of liver metastases
- At least one measurable liver metastasis according to the RECIST v1.1 criteria
- No extrahepatic disease, except pulmonary nodules if ≤3 and <10 mm, and primary tumor
- No prior chemotherapy for metastatic disease (except perioperative chemotherapy if last cycle was administered at least 12 months ago)
- Adjuvant chemotherapy after primary resection is allowed if last cycle was administered at least 12 months ago
- Normal liver function, ie, bilirubin <25 μmol/L, AST< 5N, ALT < 5N, PAL< 3N
- TP> 60%, TCK <1.5 control
- Adequate hematologic function, ie, PNN >1500, platelets >100000, Hb >9 g/dL
- Good renal function, ie, creatinine clearance >50 mL/min,
- No cardiac failure, no cardiovascular event within 6 months, NYHA ≥2

*Exclusion:*

- Patient eligible for curative treatment (ie, resection and/or radiofrequency ablation, according to the multidisciplinary committee)
- Patients unable to give their consent
- Pregnancy or breastfeeding woman, no contraceptive use in premenopausal woman
- Ongoing other cancer (except IS carcinoma of the cervix or basocellular carcinoma) Another cancer in the medical history is authorized if cured for more than 5 years
- Peripheral neuropathy
- Inflammatory bowel disease
- Bowel obstruction
- Chronic liver disease
- Allergy to contrast media that cannot be managed with standard care (eg, steroids)
- Previous organ transplantation, HIV or other immunodeficiency syndromes
- Patients with prior contraindications for the use of fluorouracil, oxaliplatin, leucovorin, and irinotecan.
- Any contraindication for hepatic embolization procedures:
  - Large shunt as determined by the investigator
  - Severe atheromatosis
  - Hepatofugal blood flow
  - Main portal vein occlusion (eg, thrombus or tumor)

1. **Chemoembolization procedure**

*Procedure*

Using a unilateral femoral approach, selective catheterization of the hepatic artery is performed. Vascular access is obtained via the common femoral artery and a guidewire advanced under fluoroscopic guidance. A 5-F sheath is then inserted over the guidewire. The superior mesenteric artery is selected and an angiogram performed to identify any aberrant arterial anatomy and verify anterograde portal vein flow. The celiac axis is then selected and an angiogram completed. The catheter and guidewire are used to select the proper hepatic artery and a limited angiogram is performed to identify the branches of the hepatic artery. The right or left hepatic artery is selected distal to the cystic artery (if visualized), depending on the location of the lesions to be treated. At the discretion of the interventional radiologist, extrahepatic vessels may be prophylactically embolized in order to mitigate the risk of non-target deposition of irinotecan beads.

For multifocal disease or tumors spanning both lobes (bilobar disease), embolization of each lobe is performed separately in two different sessions. This does not require placement of the catheter in the proper hepatic artery. The catheter should be advanced in the right or left hepatic artery. Once the catheter is in place, the irinotecan beads are delivered into the artery. Slow injection of irinotecan-loaded DC Bead® (in 1 mL aliquots followed by saline over an approx. 3-5 min period). Lipiodol should not be used with DC Bead®.

The objective of the embolization procedure is to deliver the entire dose of irinotecan (1 vial, 100 mg irinotecan) of 100-300 μm beads. It should be noted that the average volume of irinotecan DC Bead® that can be injected per treatment in colorectal patients is one vial (2 mL). The embolization endpoint is until forward flow is reduced, but avoiding backward flow of embolic agent which could embolize vessels outside the liver. No additional embolic agent should be used to achieve the embolization endpoint. Radiological non-ionic contrast (preferably Omnipaque) is used to guide the injection of beads. The catheter is then removed and hemostasis achieved by manual compression. Each patient is admitted for overnight care. The amount of contrast agent delivered to the patient during the procedure and dose of irradiation is recorded as is the time of exposure to fluoroscopic imaging. The embolized vessels are noted and the amount of embolic agent used is recorded. All medications used during the procedure are recorded, including the pain management regime.

1. **Periprocedural medication:**

Prophylactic treatment to prevent renal failure: intravenous hydration, started on the day before the procedure and continued on days 0, +1, +2 with a bag of 2000 mL per day (1000 mL of saline solution, 1000 mL of 5% glucose).

Prophylactic treatment to prevent gastric toxicity: esomeprazol 80 mg on days 0, +1, +2, infused for 24 h.

Prophylactic treatment against nausea and vomiting: ondansetron 8 mg, before the procedure, and 8 mg after 6 hours on day 0 and prednisolone 40 mg twice a day on days 0, +1, and then 40 mg/d on days +2, +3, +4, +5.

Prophylactic treatment against pain: morphine 10 mg, 30 min before and 6 h after the procedure. This prophylactic protocol is given as an indication and could be modified according to the investigator’s discretion. Intraarterial lidocaine 5 mL infused immediately before the DEBIRI procedure.

Prophylactic treatment against infection: cefazolin 2000 mg twice a day on days 0, +1, +2.

Primary prophylaxis with GSCF is recommended until all intra-arterial procedures have been completed.

1. **Definition of evaluation criterion**

OS was defined as the time between inclusion and death (whatever the cause) or the date of last news. PFS was defined as the time from inclusion to first disease progression or death (whatever the cause). Patients alive without disease progression were censored at the last follow-up visit. Survival analyses were done using the Kaplan-Meier method and described using median survivals and rates at different time points with their 95% two-sided confidence interval (CI).

Tumor response (RECIST v1.1) was assessed by investigators using chest-abdomen-pelvis CT scans every 8 weeks (4 cycles). ORR was defined as the rate of patients with complete or partial response during treatment, according to the investigator using RECIST 1.1 criteria. The secondary resectability rate was defined as the percentage of patients who underwent surgery of their hepatic metastases during or after the protocol treatment. Depth of response according to RECIST 1.1 criteria was defined as the percentage of the difference between the sum total of the largest diameters of target lesions at the nadir (in the absence of new lesions or progression of non-target lesions) and the sum total of the largest diameters of target lesions at inclusion. Early tumor shrinkage at 8 weeks was defined as the relative difference between the sum of the largest diameters of the target lesions at 8 weeks and the sum of different targets at inclusion according to RECIST 1.1 criteria. Tumor shrinkage corresponds to a relative difference >20%.

**Authors appendix :**

**Lledo G.** (Institut de Cancérologie, Hôpital privé Jean Mermoz, Lyon, FR); **Desrame J.** (Institut de Cancérologie, Hôpital privé Jean Mermoz, Lyon, FR); **Netter-coti J.** (Département de Gastroentérologie et d’oncologie digestive, Hôpital Européen George Pompidou, Paris, FR); **Landi B.** (Département de Gastroentérologie et d’oncologie digestive, Hôpital Européen George Pompidou, Paris, FR); **Ferru A.** (Service d'hépato-gastro-entérologie, CHU Poitiers, et Université de Poitiers, Poitiers, FR); **Haineaux P-A**. (Service d'hépato-gastro-entérologie, CHU Poitiers, et Université de Poitiers, Poitiers, FR); **Dermeche S.** (Oncologie médicale, IPC Institut Paoli Calmette, Marseille, FR); **Raoul J-L.** (Oncologie médicale, IPC Institut Paoli Calmette, Marseille, FR); **Oziel Taieb S.** (Oncologie médicale, IPC Institut Paoli Calmette, Marseille, FR); **Couteau C.** (Service d’HGE, CHU Toulouse Rangueil, Toulouse, FR); **Michel P.** (Hôpital universitaire de Rouen, Normandie université, service d'hépato-gastroentérologie, UNIROUEN, Inserm 1245, IRON group, Rouen, FR); **Duluc M.** (Service d'HGE et d'oncologie, CHU La Timone, Marseille, FR).

**Supplementary table S1 : post progression treatment**

| **Post-progression treatment (N=49)** |  |
| --- | --- |
| Median number of post-progression line (range) | 2 (0-7) |
| **Post progression treatment** |  |
| Anti-EGFR for RAS WT patients * | 70%* |
| Anti-VEGF | 84% |
| Irinotecan-based chemotherapy | 90% |
| Oxaliplatin-based chemotherapy | 41% |
| Regorafenib | 10% |
| TAS-102 | 18% |
| DEBIRI | 22% |

* In 20 RAS wild type patients

**Supplementary table S2 : Univariate and multivariate analysis of prognostic factors for PFS**

| Prognostic Factor |  | N Event | % Event | HR [95% CI] - p-value  Univariate analysis | HR [95% CI] - p-value  **Multivariate** analysis |
| --- | --- | --- | --- | --- | --- |
| Age | ≤ 65 years | 29/33 | 87.88 | **0.58 [0.33, 1.00] - p=0.0512** | 0.79 [0.42-1.49] – p=0.46 |
|  | > 65 years | 24/24 | 100.00 |  |  |
| ECOG PS | 0 | 22/24 | 91.67 | 0.78 [0.45, 1.37] - p=0.3875 |  |
|  | 1-2 | 31/33 | 93.94 |  |  |
| Gender | Female | 25/25 | 100.00 |  |  |
|  | Male | 28/32 | 87.50 | 0.62 [0.36, 1.07] - p=0.0854 | 0.94 [0.49-1.79] – p=0.85 |
| Molecular status | ND | 2/2 | 100.00 |  |  |
|  | BRAF mutated | 2/2 | 100.00 |  |  |
|  | RAS mutated | 29/30 | 96.67 | **0.12 [0.03, 0.58] - p=0.0083** | **0.17 [0.03-0.97] p=0.046** |
|  | RAS/BRAF wild-type | 20/23 | 86.96 | **0.07 [0.01, 0.34] - p=0.0010** | **0.08 [0.01-0.50] – p=0.007** |
| Sidedness | ND | 4/4 | 100.00 |  |  |
|  | Right colon | 12/12 | 100.00 |  |  |
|  | Left colon + rectum | 37/41 | 90.24 | 0.82 [0.42, 1.58] - p=0.5460 |  |
| Number of LM | . | 11/11 | 100.00 |  |  |
|  | ≤ 4 | 11/12 | 91.67 | 0.88 [0.44, 1.76] - p=0.7139 |  |
|  | > 4 | 31/34 | 91.18 |  |  |
| Extrahepatic M | Yes | 17/19 | 89.47 | 0.85 [0.48, 1.52] - p=0.5823 |  |
|  | No | 36/38 | 94.74 |  |  |
| Administration modality | bilobar | 19/21 | 90.48 | 0.86 [0.49, 1.52] - p=0.6084 |  |
|  | unilobar | 34/36 | 94.44 |  |  |
| Köhne index | low | 44/47 | 93.62 | 0.25 [0.06, 1.06] - p=0.0595 | 0.45 [0.10-2.11] – p=0.31 |
|  | high | 2/2 | 100.00 |  |  |
|  | Intermediate | 7/8 | 87.50 | 0.27 [0.05, 1.38] - p=0.1171 | 0.61 [0.11-3.47] p=0.58 |
| CEA | ≤ 5 ULN | 15/18 | 83.33 | **0.38 [0.19, 0.76] - p=0.0058** | **0.39 [0.19-0.81] p=0.01** |
|  | > 15 ULN | 23/23 | 100.00 |  |  |
|  | [5; 15 ULN] | 15/16 | 93.75 | 0.68 [0.34, 1.33] - p=0.2586 | 0.92 [0.40-2.09] - p=0.83 |

**Supplementary Table S3 :** **Univariate and multivariate analysis of prognostic factors for OS**

| Prognostic Factor |  | N Event | % Event | HR [95%CI] - p-value  Univariate analysis | HR [95% CI] - p-value  **Multivariate** analysis |
| --- | --- | --- | --- | --- | --- |
| Age | ≤ 65 years | 15/33 | 45.45 | **0.44 [0.21, 0.93] - p=0.0308** | 0.83 [0.31, 2.22] – p=0.71 |
|  | > 65 years | 16/24 | 66.67 |  |  |
| ECOG PS | 0 | 12/24 | 50.00 | 0.75 [0.36, 1.57] - p=0.4461 |  |
|  | 1-2 | 19/33 | 57.58 |  |  |
| Gender | Female | 17/25 | 68.00 |  |  |
|  | Male | 14/32 | 43.75 | **0.56 [0.28, 1.14] - p=0.1109** | 0.66 [0.27, 1.6] – p=0.35 |
| Molecular status | ND | 2 | 100.00 |  |  |
|  | BRAF mutated | 2/2 | 100.00 |  |  |
|  | RAS mutated | 19/30 | 63.33 | **0.08 [0.01, 0.46] - p=0.0051** | **0.14 [0.02, 1.05] – p=0.056** |
|  | RAS/BRAF wild-type | 8/23 | 34.78 | **0.02 [0.00, 0.15] - p=0.0001** | **0.05 [0.005, 0.44] – p=0.007** |
| Sidedness | ND | 4 |  |  |  |
|  | Right colon | 8/12 | 66.67 |  |  |
|  | Left colon + rectum | 20/41 | 48.78 | 0.84 [0.37, 1.92] - p=0.6839 |  |
| Number of LM | . | 11 |  |  |  |
|  | ≤ 4 | 6/12 | 50.00 | 0.91 [0.36, 2.31] - p=0.8400 |  |
|  | > 4 | 17/34 | 50.00 |  |  |
| Extrahepatic M | Yes | 8/19 | 42.11 |  |  |
|  | No | 23/38 | 60.53 | **0.57 [0.25, 1.28] - p=0.1713** | **0.17 [0.04, 0.81] – p=0.025** |
| Administration modality | bilobar | 12/21 | 57.14 | 0.92 [0.45, 1.91] - p=0.8283 |  |
|  | unilobar | 19/36 | 52.78 |  |  |
| Köhne index | low | 23/47 | 48.94 | **0.23 [0.05, 0.99] - p=0.0478** | 0.86 [0.16, 4.7] – p=0.86 |
|  | high | 2/2 | 100.00 |  |  |
|  | Intermediate | 6/8 | 75.00 | 0.44 [0.09, 2.23] - p=0.3193 | 6.46 [0.65, 63.85] – p=0.11 |
| CEA | ≤ 5 ULN | 5/18 | 27.78 | **0.30 [0.11, 0.83] - p=0.0205** | 0.36 [0.12, 1.12]  p=0.0773 |
|  | > 15 ULN | 16/23 | 69.57 |  |  |
|  | [5; 15 ULN] | 10/16 | 62.50 | 0.64 [0.28, 1.44] - p=0.2787 | 0.68 [0.23, 2.02]  p=0.4872 |

**Supplementary Table S4 : Safety**

|  | All patients (n = 57) | | |  |
| --- | --- | --- | --- | --- |
| Toxicity | Grade 1/2 | Grade 3 | Grade 4 | Grade 5 |
| **All** | **57 (100)** | **38 (66.7)** | **7 (12.3)** | **1 (1.8)** |
| **Any non-hematologic** | **55 (96.5)** | **27 (47.4)** | **2 (3.5)** | **1 (1.8)** |
| **Non-hematologic** |  |  |  |  |
| Hypersensitivity | 3 (5.3) | 0 | 0 | 0 |
| Anorexia | 14 (24.6) | 0 | 0 | 0 |
| Dysgeusia | 5 (8.8) | 0 | 0 | 0 |
| Nausea | 29 (50.9) | 3 (5.3) | 0 | 0 |
| Vomiting | 14 (24.6) | 3 (5.3) | 0 | 0 |
| Diarrhea | 24 (42.1) | 7 (12.3) | 0 | 0 |
| Abdominal pain | 11 (19.3) | 8 (14.0) | 0 | 0 |
| Small bowel obstruction | 0 | 1 (1.8) | 1 (1.8) | 0 |
| Mucositis | 11 (19.3) | 0 | 0 | 0 |
| Asthenia | 40 (70.2) | 3 (5.3) | 0 | 0 |
| Peripheral neuropathy | 46 (80.8) | 3 (5.3) | 0 | 0 |
| Alopecia | 14 (24.6) | 0 | 0 | 0 |
| Thromboembolic event | 2 (3.5) | 1 (1.8) | 0 | 0 |
| Acute coronary syndrome | 1 (1.8) | 0 | 0 | 0 |
| Hypertension | 2 (3.5) | 5 (8.8) | 1 (1.8) |  |
| Hand-foot syndrome | 8 (14.1) | 0 | 0 | 0 |
| Infection | 6 (10.5) | 2 (3.5) | 1 (1.8) | 0 |
| Peritonitis | 0 | 0 | 0 | 1 (1.8) |
| Pancreatitis | 2 (3.5) | 4 (7) | 1 (1.8) | 0 |
| Cholecystitis | 2 (3.5) | 3 (5.3) | 0 | 0 |
| **Hematologic** |  |  |  |  |
| Anemia | 53 (93) | 3 (5.3) | 0 | 0 |
| Thrombocytopenia | 33 (57.9) | 2 (3.5) | 1 (1.8) | 0 |
| Lymphopenia | 12 (21.1) | 2 (3.5) | 0 | 0 |
| Leucopenia | 11 (19.3) | 1 (1.8) | 0 | 0 |
| Neutropenia | 22 (38.6) | 8 (14) | 6 (10.5) | 0 |
| Febrile neutropenia | 5 (8.8) | 1 (1.8) | 2 (3.5) | 0 |

**Supplementary Table S5 : Safety per procedure (occurring during the first 24 h after the DEBIRI session) and per patient, and according to the lobar modality of administration of DEBIRI**

| n= number of embolizations  N= number of patients | All patients  n=142 (N=57, %) | | Unilobar  n=103 (N=36 patients, %) | | Bilobar  n = 39 (N=21 patients, %) | |
| --- | --- | --- | --- | --- | --- | --- |
|  | **n=146 (%)** | **N=57 (%)** | **n=103 (%)** | **N=36 (%)** | **n=39 (%)** | **N=21 (%)** |
| **Post-embolization syndrome** | **76 (53.5)** | **46 (80.7)** | **54 (52.4)** | **31 (86.1)** | **22 (56.4)** | **15 (71.4)** |
| Nausea/vomiting | 16 (11.2) | 13 (22.8) | 12 (11.7) | 9 (25) | 4 (10.3) | 4 (19) |
| Pain: visual analog scale >3 | 67 (47.2) | 43 (75.4) | 48 (46.6) | 29 (80.6) | 19 (48.7) | 14 (66.7) |
| Hyperthermia | 3 (2) | 3 (5.3) | 3 2.9) | 3 (8.3) | 0 | 0 |
| Hypothermia | 4 (2.8) | 3 (5.3) | 3 2.9) | 2 (5.6) | 1 (2.6) | 1 (4.8) |
| **Cardiovascular event** | **22 (15.5)** | **15 (26.3)** | **13 (12.6)** | **9 (25)** | **9 23.1)** | **6 (28.6)** |
| Hypertension | 13 (9.2) | 11 (19.3) | 6 (5.8) | 6 (16.7) | 7 (17.9) | 5 (23.8) |
| Thoracic pain | 7 (4.9) | 3 (5.3) | 5 (4.9) | 2 (5.6) | 2 (5.1) | 1 (4.8) |
| Tachycardia | 2 (1.4) | 2 (3.5) | 2 (1.9) | 2 (5.6) | 0 | 0 |
